# The Inexorable Spread of a Newly Arisen Neo-Y Chromosome

**DOI:** 10.1371/journal.pgen.1000082

**Published:** 2008-05-30

**Authors:** Paris Veltsos, Irene Keller, Richard A. Nichols

**Affiliations:** School of Biological and Chemical Sciences, Queen Mary University of London, London, United Kingdom; University of Aarhus, Denmark

## Abstract

A newly arisen Y-chromosome can become established in one part of a species range by genetic drift or through the effects of selection on sexually antagonistic alleles. However, it is difficult to explain why it should then spread throughout the species range after this initial episode. As it spreads into new populations, it will actually enter females. It would then be expected to perform poorly since it will have been shaped by the selective regime of the male-only environment from which it came. We address this problem using computer models of hybrid zone dynamics where a neo-XY chromosomal race meets the ancestral karyotype. Our models consider that the neo-Y was established by the fusion of an autosome with the ancestral X-chromosome (thereby creating the Y and the ‘fused X’). Our principal finding is that sexually antagonistic effects of the Y induce indirect selection in favour of the fused X-chromosomes, causing their spread. The Y-chromosome can then spread, protected behind the advancing shield of the fused X distribution. This mode of spread provides a robust explanation of how newly arisen Y-chromosomes can spread. A Y-chromosome would be expected to accumulate mutations that would cause it to be selected against when it is a rare newly arrived migrant. The Y can spread, nevertheless, because of the indirect selection induced by gene flow (which can only be observed in models comprising multiple populations). These results suggest a fundamental re-evaluation of sex-chromosome hybrid zones. The well-understood evolutionary events that initiate the Y-chromosome's degeneration will actually fuel its range expansion.

## Author Summary

Comparisons between related species have shown that, over evolutionary time scales, Y-chromosomes tend to degenerate and can be completely lost. How then can we explain the persistence of Y-chromosomes to the present? One possibility is that losses are counter-balanced by the origin of new Y chromosomes, which then spread throughout the species in which they have arisen. The first of these two processes, the generation of new Y chromsomes, is more readily understood: it can occur if an autosome (a non sex chromosome) fuses with an X chromosome. This form might become established in one locality. However, its subsequent geographic spread has been more challenging to explain. Problems arise if gene flow carries them to another part of the species range. Crosses can then occur which introduce the new Y chromosome into females, who are expected to suffer reduced fitness. The new sex chromosomes are therefore selected against when they are in the minority. We use simulations to show that they can nevertheless spread, if they meet the ancestral forms at a front so the chromosomes intermingle in a hybrid zone. Paradoxically, the degeneration of the Y will actually intensify selection, thereby speeding its spread.

## Introduction

Our understanding of sex chromosome evolution has increased immensely in the past decade. Theoretical expectations [1],[2] have been experimentally verified in a wide variety of organisms, including fish, fruitflies, mammals and plants [3]–[8]. For example, it is widely documented that over a series of generations, a Y-chromosome will eventually stop recombining with the X over most of its length. As a consequence there are increased rates of transposition, degeneration, heterochromatinization and loss of function of genes on the Y, amongst other changes [5], [8]–[13].

It appears then, that the inexorable fate of Y-chromosomes is degeneration and perhaps loss. It is even possible that all sexually dimorphic species lacking a Y have previously passed through a Y-possessing stage [1],[14], as is the case for *Caernohabditis elegans* [15] (the logic would also apply to equivalent W chromosomes in species with heterogametic females). The persistence of Y-chromosomes to the present day therefore suggests that they can repeatedly arise *de novo.* One straightforward way in which new Ys can be created is by the fusion between an autosome and X-chromosome followed by its fixation. This paper models the evolution of such neo-XY sex chromosome systems and, in particular, asks why they should become established throughout a species' range. The analysis suggests that the spread of neo-Ys is much more likely than suggested by current models, and that this new proposal could be tested by analysis of sex chromosome hybrid zones.

**Figure 1:**
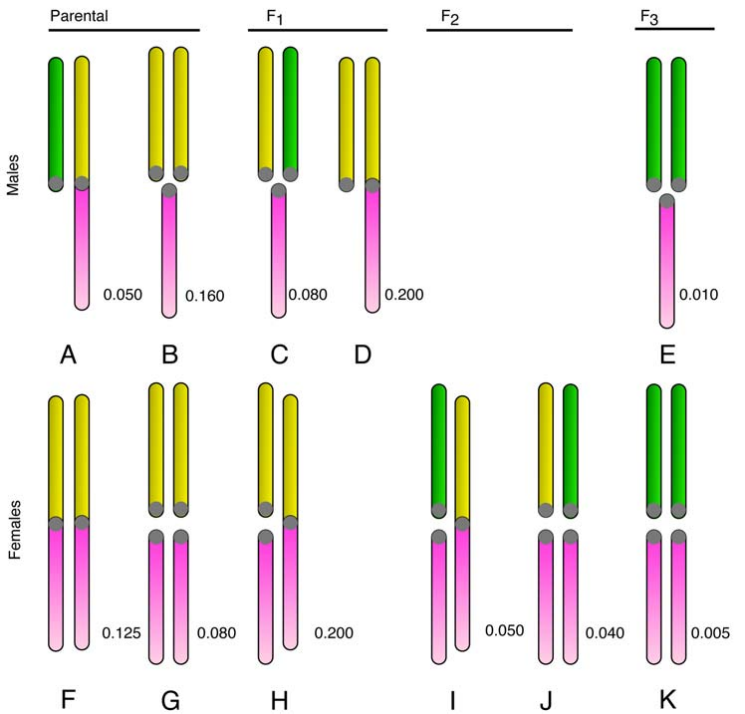
The karyotypes occurring in the hybrid zone. The karyotypes are organised by sex and the generation they first appear (in a cross between neo-XY and XO populations). The lower pink chromosome is the ancestral X. The upper chromosome can be an unfused A_u_ (yellow), or fused to the X (as part of the neo-X, also shown in yellow), or a Y-chromosome (green). Each karyotype has been labelled by a letter, for reference. The expected frequency of each karyotype at equilibrium is shown for the neutral case after a cross between equal numbers of new-XY and XO. Note the 1∶4 ratio of the Y to unfused A_u_ in the parental populations (made of A, B, F, G).

A concrete example can be a useful guide for explaining and constructing evolutionary models, so we make use of the well-studied example of the neo-XY race of the grasshopper *Podisma pedestris*. Phylogenetic comparison [16] suggests that the ancestral *P. pedestris* karyotype had females with two X-chromosomes and males with one (but no Y): this is known as an XX/XO sex-determining system [17]. The system changed following the centric fusion of the X with an autosome (A_u_) to create a larger metacentric neo-X. The fused karyotype has become fixed in populations in the southern part of the species' distribution in the French Alps. In these fixed populations, the females contain two neo-X chromosomes, and hence no unfused A_u_. In males, however, the unfused A_u_ chromosomes have continued to pair with the homologous section of the neo-X. These unfused A_u_ are now restricted to males and are consequently designated neo-Y chromosomes. The karyotypes of the original unfused, and derived fused race are illustrated in Figure 1 (karyotypes A, B, F, G).

Sex chromosomes are often involved in fusions. Indeed, human sex chromosomes are believed to be the products of at least three chromosomal fusions [6],[18],[19], as is the *Drosophila* Y-chromosome [20]. The occurrence of a fusion is insufficient to explain the genesis of a neo-XY system however. Following the fusion event, the new karyotypes must also become fixed throughout the species range (or part of it). The establishment of neo-XY systems does appear to occur repeatedly in evolution. Good evidence comes from the Orthoptera, which have conveniently large chromosomes for surveys of karyotype. White calculates that there have probably been six independent fixations of the XY system from an ancestral XO condition in the Australian subfamily Morabinae alone (based on karyotypes from about 80 species). More generally, the fixation of the XY system has been reported in at least 21 genera of Acrididae [16](and references therein).

Attempts to explain this establishment fall broadly into two traditions. Firstly, cytogeneticists have noticed that chromosomal rearrangements often confer reduced fertility in heterozygotes. The cause may be a direct effect of meiotic aberrations [21],[22]; in other cases selection against changed recombination patterns is suspected (see [23] for an example). These forms of selection actually act against the fusion when it is rare; but it could nevertheless become established in small isolated populations if genetic drift elevated its frequency until it became the commoner type and hence favoured by selection [24]. The difficulty with this explanation is to account for how the fusion would subsequently spread from a single isolated population to other populations. Lande [24] proposed that the fused race could colonize sites left vacant by local extinctions, whereas Hewitt [25] argues that spreading would be more effective if the initial population was located on the expanding margin of the species range, as it spreads into new territory–most likely during an episode of rapid climatic change.

A second perspective comes from consideration of the alleles that were segregating on autosomes before the fusion occurred. Fusion with a sex chromosome might bring alleles into linkage with the newly created sex chromosome and confer a fitness benefit; the key alleles might be sexually antagonistic (benefiting one sex at the expense of the other) [26] or deleterious recessives [27] (especially in strongly inbreeding populations). In both cases the selection is expected to be much more effective in promoting Y-autosome fusions, and such events might repeatedly add new genetic material to existing Y-chromosomes. Linkage with sexually antagonistic alleles could also produce selection for the fixation of new X-autosome fusions, and hence the creation of neo-Ys.

There is some evidence that sexually antagonistic alleles may indeed have appreciable effects. Rice conducted an imaginative breeding design in which a haploid *Drosophila* genome was restricted to one sex for several generations and then returned to the other sex [28]–[30]. The results were striking. In less than 30 generations, sex specific fitness differences had become established in the sex-restricted genome. The rapidity of the response was interpreted as showing that sexually antagonistic alleles had been segregating in the founder population.

Even with strong selection on sexually antagonistic alleles, the advantage provided to the fused chromosome would be weak [26]. Nonetheless, this selective process, or the action of drift, might establish the neo-XY system locally in part of the species distribution. The spread throughout the whole species range is more difficult to explain. Any advantage to the fusion when rare is expected to be transient, because of the well-understood evolutionary events affecting new sex chromosomes. Alleles reducing female fitness, can accumulate readily on the Y [1],[2], particularly if they also had beneficial effects in males.

Our analysis, has uncovered a paradoxical effect that nevertheless favours the geographic spread of the neo-XY system. If sexually antagonistic alleles have become established on the Y, the genetic interactions at the boundary between neo-XY and ancestral populations can favour the spread of the neo-X. Surprisingly, the results hold even if the net effect of selection against the neo-Y in females outweighs the benefits in males.

## Materials and Methods

**Figure 2:**
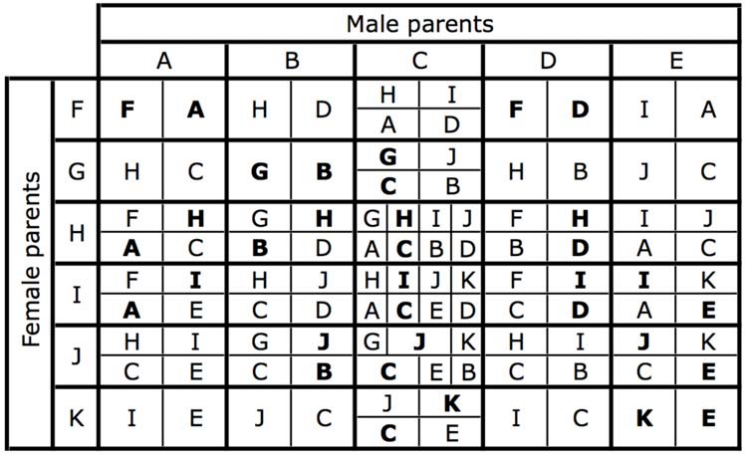
The karyotypes produced by each possible mating. The letters refer to the karyotypes in Figure 1. The area of each cell indicates the relative proportion of each karyotype in a mating. Offspring with the same karyotypes as their parents are indicated in bold. This matrix forms the basis of the computer simulations.

**Figure 3:**
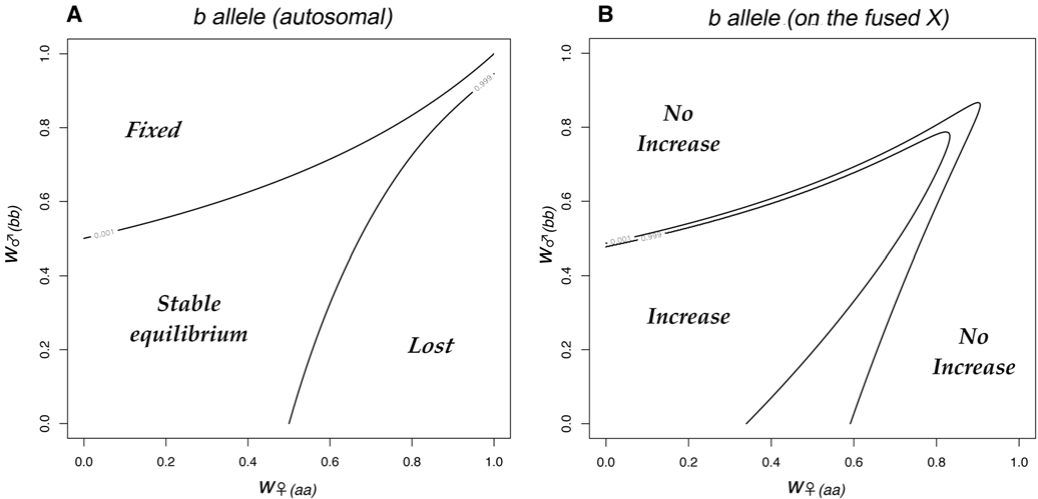
The fate of sexually antagonistic alleles in a single population. We consider an ancestral autosomal locus, which had two sexually antagonistic alleles: *a* was favoured in males and *b* in females. 3A. The outcome of selection on the *b* allele in the ancestral population as a function of the fitnesses of the two homozygotes. The central area of fitness combinations results in a stable polymorphism (delineated by the contours *p_a_ = *0.001 and *p_a_* = 0.999). In this example the alleles were additive. 3B. The evolutionary dynamics change if one of the *b*-bearing autosomes fuses to the X-chromosome. We illustrate this effect by plotting the frequency of the *b*-neo-X haplotype 1 000 generations after it has been introduced at low frequency (0.04). The neo-X spreads for some fitness combinations (the central area enclosed by 0.001 and 0.999 contours). The spread of the neo-X was opposed by weak selection against females heterozygous for the fusion (genotypes H and I from Figure 1 were assumed to suffer a 1% reduction in fertility). Note, that mild sexually antagonistic selection (i.e. the region near the point (1,1)) is insufficient to favour the fused X.

The pattern of chromosome segregation in crosses involving individuals with different sex chromosome combinations is illustrated in Figure 2. The letters correspond to the karyotypes shown in Figure 1, the area of each cell is proportional to the number of each karyotype in the offspring. This scheme was translated into a set of equations for the frequency of each karyotype as a function of the frequencies in the previous generation, assuming random mating and after weighting each karyotype by its fitness. A program to iterate the equations was written in the statistical language R [31], and is listed in the supporting information (Dataset S1). The initial analysis revisited, and then extended the results of [26]. Consider two sexually antagonistic alleles that might be segregating on the autosome A_u_ during the period before the chromosomal fusion. The two alleles (***a*** and ***b***) have different fitness in the two sexes (specified by *w_♀aa_*, *w_♀ab_* & *w_♀bb_* for females, and *w_♂aa_*, *w_♂ab_* & *w_♂bb_* for males). The ***a*** allele was assumed to be favored in males, and the ***b*** in females so *w_♀bb_* = *w_♂aa_* = 1. The program calculated the outcome of selection for all possible combinations of *w_♂bb_* and *w_♀aa_* in the range 0–1 at intervals of 1/40 (with a specified dominance). If there is polymorphism at this locus before the fusion takes place, the fusion will (in some cases) generate linkage disequilibrium leading to selection for the fixation of the fusion. The fitness combinations leading to such polymorphism can be illustrated by initiating a simulation with only the ancestral XX∶XO karyotypes, and including a chromosome carrying the ***a*** allele (equivalent to the green chromosome in Figure 1) at low frequency. Figure 3A illustrates a range of fitness combinations producing polymorphism (achieved from an initial ***a*** frequency of 0.1% with all genotypes in Hardy-Weinberg proportions).

The combinations of fitness that can lead to selection for the fixation of the fusion have been explored in some detail [26]. In Figure 3, they correspond to the ***b*** allele becoming linked to the X by the fusion. This selection for the fusion could be demonstrated in our simulations by initiating the fusion at a low frequency (0.4%, corresponding to the frequency of the fusion, if migration had introduced the Y at 0.1%) and then iterating the equations for 1000 generations. The analysis was extended to investigate the effect of the slightly reduced fertility that is expected in females heterozygous for the fusion. This additional selection was set at *s* = 0.01, the value estimated for *P. pedestris.* The recombination rate between the ***b*** allele and the X centromere was set at zero to maximize the selection for the fusion [26]. As the Y-chromosome evolves, recombination is expected to be reduced over a greater proportion of the Y [1], hence the modelled effects are increasingly likely to occur. Indeed, in the case of *P. pedestris*, which is assumed to have a young neo-XY system, the recombination is already displaced way from the Y centromere [32], perhaps simply as effect of the fusion itself, and very strong linkage disequilibrium has been found even in the middle of the zone for an X-marker [33].

### Simulation of a Contact Zone between XO and Neo-XY Populations

These initial calculations involved a single panmictic population. The outcome can be different when the population is subdivided. The next step was therefore to consider a situation in which the neo-XY system had become established in an isolated area, and come into contact with the ancestral (XX:XO) karyotype. Gene flow between the two chromosomal races would then produce a hybrid zone. A computer simulation of a linear array of 40 populations was used to model this situation. Initially the left hand 20 populations were fixed for the ancestral karyotype and the remainder for the neo-XY. There was gene flow of 8% between adjacent populations (total gene flow of 16%). Population size was uniform across the simulated populations. In other words there was no density trap to pin the zone down to a particular location (as described by [34]). The two ends of the array could either be set to receive gene flow from populations fixed for the ancestral karyotypes, or to only receive gene flow from their more central neighbour. Both options were used to check for any effect on the outcome of the simulations. For each generation, after gene flow, the expected frequencies of genotypes in the next generation were calculated as before.

In this extension of the model, the green chromosome in Figure 1 is considered to be a neo-Y-chromosome. The ***a*** allele would have been fixed on the neo-Y which could also have accumulated additional sexually antagonistic alleles tightly linked to the X centromere (due to the extension of the non-recombining region). We have argued that we would expect some alleles to be selected against in males and favoured in females, and for there to be selection against the chromosomal heterozygotes. However, it is helpful to understand the combination of these effects by first examining the behaviour of these three forms of selection individually. We therefore summarize the results by defining three selection regimes. The first two correspond to points on the X and Y axes of Figure 3: Firstly, male-beneficial variants could have become established on the Y (*w_♂(AU·)_*<1), were ‘A_u_ *·*’ represents genotypes containing A_u_– the autosomal homologue of the neo-Y; secondly, female-deleterious variants could occur on the Y (*w_♀(Y·)_*<1). The third simple case is selection against females heterozygous for the fused X (*w_♀(FU)_*<1). We then simulated all possible combinations of the fitness regimes. Table 1 sets out the karyotypes with reduced fitness in each regime.

**Table 1:** The finesses of the karyotypes under different forms of selection.

	Karyotype fitness							
	Male				Female			
	B	C	D	E	H	I	J	K
**Sexually antagonistic selection**	1−*s_m_*	1−*s_m_ d_m_*	1−*s_m_*	1	1	1−*s_f_ d_f_*	1−*s_f_ d_f_*	1−*s_f_*
**Heterozygote disadvantage**	1	1	1	1	1−*s_h_*	1−*s_h_*	1	1
**Dosage compensation**	1	1−0.5*s_d_*	1−0.5*s_d_*	1−0.5*s_d_*	1−0.5*s_d_*	1	1−0.5*s_d_*	1−*s_d_*
**Sex chromosome coadaptation**	1	1−0.5*s_c_*	1−*s_c_*	1−*s_c_*	1−0.5*s_c_*	1−0.5*s_c_*	1−0.5*s_c_*	1−*s_c_*

**Figure 4:**
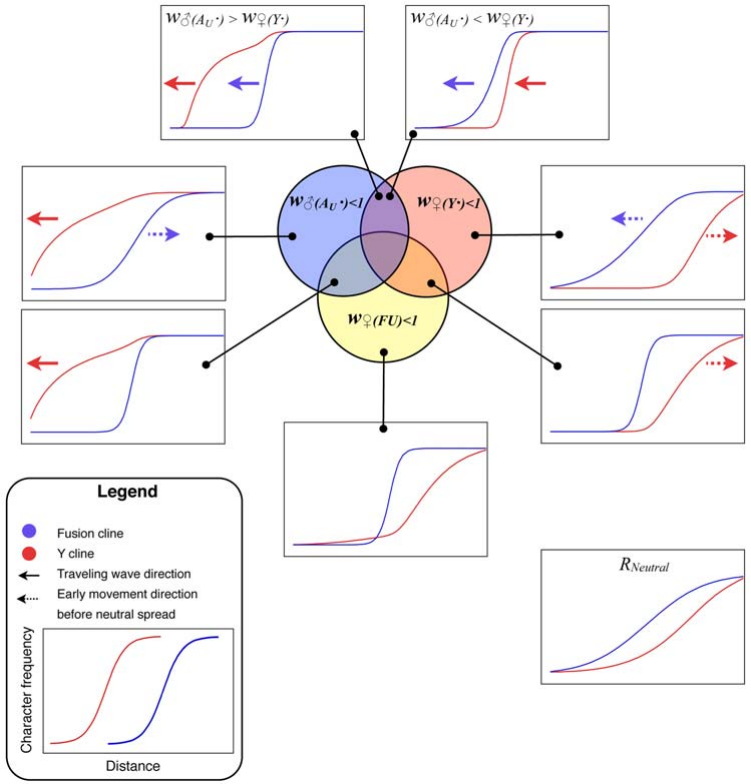
Summary of the simulation results for the Y and fusion clines. The selection is described according to the genotype with reduced fitness (*w_♂(AU·)_*, *w_♀(Y·)_*, *w_♀(FU)_*) as set out in the first row of Table 1. In the particular examples illustrated here the fitness was reduced by setting the selection coefficent (*s_m_, or s_f_)* to 0.1 (with *d_m_ = d_f_ = *1). The only exceptions are in the upper two panels, in which case the extreme selection coefficient was set to 0.9.

If more than one form of selection was acting, the values in the corresponding columns were multiplied. The forms of section shown in Figure 4 correspond to the following:

***w_♂ (AU·)_***<1≡*s_m_* = 0.1, *s_f_* = 0, *d_m_* = 1; ***w_♀(Y·)_***<1≡*s_m_* = 0.1, *s_f_* = 0, *d_m_* = 1; ***w_♀(FU)_***<1≡*s_h_* = 0.1.

Having determined the basic patterns produced by the different fitness regimes, we assessed the spread of the neo-XY system throughout the possible parameter range shown in Figure 3. The simulations were run until fixation or until 10 000 generations. We explored the full range of values for *s_f_* and *s_m_* in the presence of selection against chromosomal heterozygotes, which was set to that estimated in *P. pedestris* of *s_h_* = 0.01 (fitnesses specified in the first and second rows of Table 1, combined multiplicatively). The dominance of the sexually antagonistic selection (*d_m_* and *d_f_* in Table 1) was varied: from *d_m_ = *1 or 0.5 for the male effect (not zero since recessive male-beneficial alleles would have had no advantage on the Y), and *d_f_* = 1, 0.5 or 0 for the female effect. In addition to these forms of selection, we also considered the possibility that there had also been evolution of coadaptation or dosage compensation between the sex chromosomes in the established neo-XY and XO populations, giving rise to the fitnesses in the last two rows of Table 1.

The model zone width was converted to values that could be observed in the field using the relationship *σ* ^2^ = *mD^2^,* were *σ* ^2^ is the variance in parent-offspring dispersal and is a measure of migration. For *P. pedestris*, it has been estimated by mark-release-recapture experiments to be 400 m^2^ per generation [35]. The value *D* represents the distance in the field equivalent to that between adjacent simulated populations. Since the simulated migration rate, *m,* was 0.16, the real hybrid zone width of 800 m [34] is equivalent to the distance between 16 simulated populations.

When all fitnesses were set to one, the simulated width of the zone increased with time–matching the neutral expectation *w* = 2.51*σ√t*, where *w* is the width, *σ* is the parent-offspring dispersal per generation and *t* is time in generations [36] (results not shown). Similarly, in the case *w_♀(FU)_*<1, the simulated width of the fusion cline fitted analytical expectations, as long as the female-specific nature of selection was taken into account (see Results). The program to simulate the structured populations was written in Java and the full source is available from the authors upon request.

## Results

Figure 3B shows the neo-XY chromosomes can invade an ancestral XO population when they are introduced at low frequency, if there is strong sexually antagonistic selection (the central area of the Figure). The spread of the fused X (the neo-X) was accompanied by fixation of the ***a*** allele. The model included weak selection against females heterozygous for the fusion, hence the fusion was selected against when rare. Consequently there were combinations of low to moderate selection (i.e. around (1,1)) for which the fusion did not spread. This failure to spread occurred irrespective of the dominance of the ***a*** allele in males, or the ***b*** allele in females (results not shown).

Figure 4 summarizes the various outcomes of the simulated meeting between the two chromosomal races to form a hybrid zone. The relative frequency of the fusion has been plotted as against distance along the array of populations. This relative frequency was calculated as *f_f_/(f_f_ + f_u_)*, where *f_i_* specifies the frequency of the chromosome of type *i*∈{f, u, Y, Au}, representing fused X, unfused X, Y and autosome respectively. Similarly, the frequency of the Y was calculated as *f_Y_/(f_Y_ + f_Au_)*. Note that the denominator increases with *f_u_*, since unfused individuals carry more of these chromosome (i.e. more Au and/or Ys, see Figure 1). Once the two races meet, gene flow produces a sigmoidal transition in the frequency of the fused X and the Y-chromosomes. For some parameter values the Y cline or the fusion cline spread as a wave of advance, indicated by arrows in Figure 2, in other cases the clines were stable or decayed (see Discussion).

Existing analytical models do not describe much of this behaviour, but there are well known results for the case of heterozygote disadvantage *w_♀(FU)_*<1 under which the fusion cline would assume a fixed position and width. The relationship between the strength of selection (in the range 0.05–0.9) and width (the inverse of the maximum slope [37]) closely fitted the expected relationship *w = √*8*σ/√s* [38] as long as the selection coefficient, *s*, was multiplied by 2/3 to compensate for selection acting on females only (r^2^>99.99%, regression coefficient = 1.016).

The examples in Figure 4, in which the neo-XY system spreads (upper two panels), involve strong sexually antagonistic selection–chosen to clearly illustrate the qualitative difference in outcome from single population models (in which it did not for these values). The neo-XY system spreads even when there is strong selection against the Y in females.

We investigated the rate of spread of the neo-XY system under less severe selection (Table 2). Selection against the X chromosome heterozygotes of 1% (*s_h_* = 0.01) slowed down the rate of spread slightly (up to 50%), but did not prevent it even when the sexually antagonistic selection was weak (e.g. *s_m_ = s_f_* = 0.005). Other forms of selection against introgression could both accelerate or retard the rate of spread. We investigated the fitnesses that might be generated by coadaptation and dosage compensation of the sex chromosomes (Table 1). Both forms of selection accelerated the spread when the selection for the Y in males was greater than the disadvantage in females (*s_m_*>*s_f_*) and retarded or even slightly reversed the direction of spread under the converse (*s_m_*<*s_f_*) (Table 2). The effect of dominance was minor over most fitness combinations and the outcomes were qualitatively unaffected. We show the effects of male dominance in Table 2, but omit the female for brevity.

**Table 2:** The speed of the fusion cline's advance.

***D_m_*** ** = ** ***D_f_*** ** = 0.5**								***D_m_*** ** = 1, ** ***D_f_*** ** = 0.5**							
		***S_m_***								***S_m_***					
		**0.005**	**0.01**	**0.02**	**0.05**	**0.15**	**0.9**			**0.005**	**0.01**	**0.02**	**0.05**	**0.15**	**0.9**
***S_f_***	**0.005**	2500	1950	1600	1400	1300	1300	***S_f_***	**0.005**	2400	1800	1350	1000	900	800
	**0.01**	2450	1850	1350	1050	950	500		**0.01**	2500	1850	1350	850	650	550
	**0.02**	2600	1950	1350	850	650	600		**0.02**	2600	2000	1400	850	500	350
	**0.05**	2700	2150	1550	850	450	400		**0.05**	2700	2150	1600	950	450	200
	**0.15**	2750	2300	1750	1050	450	200		**0.15**	2750	2300	1750	1100	550	50
	**0.9**	2800	2400	1900	1250	650	100		**0.9**	2800	2400	1900	1250	650	100
***S_h_*** ** = 0.01**		***S_m_***						***S_h_*** ** = 0.01**		***S_m_***					
		**0.005**	**0.01**	**0.02**	**0.05**	**0.15**	**0.9**			**0.005**	**0.01**	**0.02**	**0.05**	**0.15**	**0.9**
***S_f_***	**0.005**	5600	3600	2900	2600	2500	2400	***S_f_***	**0.005**	5750	3200	2100	1750	1850	1600
	**0.01**	5950	3200	2050	1550	1400	1350		**0.01**	5850	3350	1900	1150	1000	900
	**0.02**	5900	3500	1900	1050	850	800		**0.02**	5750	3500	2050	1000	600	500
	**0.05**	5600	3650	2200	1000	500	450		**0.05**	5500	3600	2200	1100	500	200
	**0.15**	5500	3750	2400	1300	500	200		**0.15**	5500	3700	2400	1300	550	100
	**0.9**	5850	3950	2600	1450	700	100		**0.9**	5850	3900	2600	1450	650	100
***S_d_*** ** = 0.01**		***S_m_***						***S_d_*** ** = 0.01**		***S_m_***					
		**0.005**	**0.01**	**0.02**	**0.05**	**0.15**	**0.9**			**0.005**	**0.01**	**0.02**	**0.05**	**0.15**	**0.9**
***S_f_***	**0.005**	6050	2800	1550	850	600	550	***S_f_***	**0.005**	6450	3050	1700	900	550	300
	**0.01**	7550	3150	1650	850	550	500		**0.01**	7900	3350	1800	950	500	300
	**0.02**	20000	3750	1850	850	500	450		**0.02**	20000	3900	2000	1000	500	250
	**0.05**	32500	5100	2350	1000	450	300		**0.05**	30650	5100	2400	1100	500	150
	**0.15**	−100000	7350	3000	1300	500	200		**0.15**	−100000	7250	3000	1350	550	100
	**0.9**	−13500	10950	3750	1600	700	100		**0.9**	−33350	15400	3750	1600	700	100
***S_c_*** ** = 0.01**		***S_m_***						***S_c_*** ** = 0.01**		***S_m_***					
		0.005	0.01	0.02	0.05	0.15	0.9			0.005	0.01	0.02	0.05	0.15	0.9
***S_f_***	**0.005**	−66700	4850	1900	900	600	550	***S_f_***	**0.005**	−66700	5450	2200	1000	550	300
	**0.01**	−33300	5850	2100	900	550	500		**0.01**	−13350	6400	2350	1000	500	300
	**0.02**	−12200	8100	2450	950	500	450		**0.02**	−22250	8500	2650	1100	500	250
	**0.05**	−5500	−33300	6550	1850	650	100		**0.05**	−5600	−33300	6400	1800	650	100
	**0.15**	−6300	−100000	4800	1500	500	200		**0.15**	−6400	−200000	4700	1500	600	100
	**0.9**	−5400	−25000	7250	1950	700	100		**0.9**	−5500	−14000	7100	1900	700	100

The speed is expressed as the number of generations for the fusion cline to advance 1 Km. The location of the cline centre was output every 50 generations, and speed was estimated as the time taken to reach the end of the array (equivalent to 1 Km in *P. pedestris*). In those cases where the end was not reached within 10000 generations, the speed was estimated from the movement to that time.

## Discussion

In the Introduction we outlined how the fixation of an X-autosome fusion could be explained by selection in favour of sexually antagonistic alleles linked to the fused centromere. Charlesworth and Charlesworth [26] have shown that there is net selection in favor of the fusion for fitness combinations that lead to polymorphism at the sexually antagonistic locus: which fall in the central shown in Figure 3A. However, Figure 3B suggests that this form of selection might be readily counteracted, even by a very minor (1%) reduction in the fertility of female fusion heterozygotes, *w*_♀*(FU)*_. In particular, even relatively strong sexually antagonistic selection is overwhelmed: notice that when this additional selection is applied (Figure 3B) the fusion does not spread for fitness values within 0.15 of the point (1,1) (i.e. selection coefficients of up to 15%) even if they fall within the polymorphic area in Figure 3A. Selection against female fusion heterozygotes is considered likely because of non-disjunction at meiosis [21], and is indeed suspected to occur in *P. pedestris* [37]. In these circumstances, it is easier to envisage the fusion becoming established by genetic drift, than deterministically under the action of selection.

Whatever the reason for the fusion initially becoming established in one locality, once it is fixed, the A_u_ autosome will then be restricted to males, and would consequently have become a neo-Y. The subsequent evolution of the sex chromosomes would therefore take a course that would at first sight seem to make the spread of the neo-XY system even more unlikely. In particular the neo-Y is expected to accumulate further sexually antagonistic effects, which would in turn select for the loss of recombination, and its eventual degeneration [1]. The selection acting would therefore be stronger (further away from the point (1,1) in Figure 3B) and for the most part this would lead to even stronger selection to eliminate the neo-XY system: only if selection is strong and of similar order in males and females (the triangular area) would selection favour its spread.

The model illustrated in Figure 3B assumes a small starting number of neo-XY individuals. Remarkably, the outcome is completely overturned if the XO and neo-XY populations are assumed to meet in a hybrid zone. The spread of the neo-XY system would actually be driven by the selection regimes that lead to its elimination in Figure 3B.

### Understanding the Simulations of a Hybrid Zone

It may be simplest to start interpreting the results using the biologically unrealistic case of selection only against females containing Y-chromosomes, *w*_♀*(Y·)*_<1. Since only females with an unfused X chromosome can contain a Y, this regime leads to selection against them, causing the fusion cline to advance (Figure 4, *w*_♀*(Y·)*_<1). However, in the absence of other selection, this effect is transient since the direct selection on the Y removes it from populations containing unfused chromosomes. In other words, the autosome (A_u_) advances because it is favoured by selection. The Y persists only in the heartlands of the fused chromosome range, because there it experiences no disadvantage because it cannot enter females.

There is a comparable indirect effect on the fusion in the case of selection only in favour of Y-chromosomes in males, *w*_♂*(Au·)*_<1. In populations that are polymorphic (for Y/A_u_), unfused males are more likely to contain at least one advantageous Y because they have double the number of these chromosomes (in fused males, the X replaces one of them). Hence *w*_♂*(Au·)*_<1 results in selection against the fusion (Figure 4, *w*_♂*(Au·)*_).

We can extend these explanations to the most interesting and biologically relevant result–the wave of advance for both the Y and the fusion clines under sexually antagonistic selection (*w*_♂*(Au·)*_<1 & *w*_♀*(Y·)*_<1). When the selection against the Y in females is stronger, the Y-chromosome tends to be removed from the fusion cline as under *w*_♀*(Y·)*_<1. However, as the fusion advances (for the same reason as under *w*_♀*(Y·)*_<1) the Y follows behind, up to the margins of the fusion cline, thereby indefinitely maintaining the selection for the advance of the fusion. Note that this neo-XY success depends on the gene flow continually bringing the Y-chromosomes into the zone, which is why it did not occur in the single partially isolated population of Figure 3B.

The neo-XY system also spread when there is a net advantage to the Y (under sexually antagonistic selection). However, in this case, the Y spreads as a traveling wave ahead of the fusion cline. The fused X will also spread because, as the Y becomes common, the selection against unfused females gets stronger whereas the benefit to unfused males is reduced (since both fused and unfused males tend to carry the favourable Ys once they become common).

The observed speed of spread of the fusion was relatively small compared to dispersal: the fastest being equivalent to 450 generations to move 1 km in *P. pedestris*, or 1/10th of the dispersal distance per generation. It would be difficult to observe by repeated sampling, but would cause consistent movement over evolutionary time. In addition, the sexually antagonistic effects could increase in magnitude with time, because newly evolved sexually antagonistic alleles arising in within the neo-XY range would be expected to spread until they met the hybrid zone.

We consider the forms of dominance in Table 2 to be most likely, although we have explored other combinations and found no qualitatively different outcomes. Dominant male beneficial effects would fix faster initially, making them more likely to evolve, however such alleles could spread in XO or neo-XY populations, so they need not be restricted to the neo-XY race; hence there could be a range of dominance for male beneficial effects. The first female deleterious effects on the Y would probably be due to loss of function mutations, and would therefore be less than fully dominant because the functional ancestral allele would still be present.

In addition to heterozygote disadvantage we also considered alternative forms of selection that might act against recombinant karyotypes in the hybrid zone. As long as these additional effects are of the same order as the sexually antagonistic selection, the results are essentially unchanged. It is, of course, possible to find some fitness combinations that slow down, or even slight reverse the spread, and it could be counter-acted by assymetrical gene flow (e.g. due to a density gradient [34]) or other selection (e.g. due to change in the environment). However, Rice's experiments [28]–[30] suggest that the alleles with large sexually antagonistic effects are segregating in natural populations, so we would expect their effects to predominate in the zone as soon as it was formed. If this interpretation is correct, more detailed analysis of hybrid zones should provide additional evidence of this sexually antagonistic selection (see below).

### Strong Selection Can Produce Broad Clines

Interestingly, strong selection on the Y chromosome resulted in a broad fusion cline (Figure 4). The result emphasizes that the width of the cline in the character for which the hybrid zone was originally discovered, need not indicate the strength of selection. In fact the term “hybrid zone” can be misleading in these cases; it is preferable to refer to different clines. The broad fusion cline in the presence of strong selection is particularly relevant to the *P. pedestris* hybrid zone, where strong selection is detected in the F_1_ in lab crosses [37],[39] and in the field [40], yet the fusion cline is much wider than expected from the observed selection [41]. Previously, this discrepancy has been explained by a model in which selection is spread over many loci [41] only some of which need be linked to the fusion, but our results offer an alternative possibility: that the action of the selection is indirect and due to the well understood initial events in sex chromosome evolution.

### Sex Chromosome Hybrid Zones as Natural Sex Chromosome Evolution Experiments

One implication of the results is that sex chromosome hybrid zones are a valuable, yet unexploited, source of information on early sex chromosome evolution. We suggest that it will be rewarding to obtain markers that distinguish the Y-chromosome from its homologous autosome (A_u_ in our notation) and to survey their geographic distribution across known sex chromosome hybrid zones. Often the clines of different characters coincide (have the same centre) [42],[43], however we would expect them to be displaced in the case of sexually antagonistic selection. Comparing the Y and the fusion cline as in Figure 4 using real hybrid zone data is a robust way to identify the selection regime operating.

The conventional explanation for a narrow sex chromosome hybrid zone is that there is selection against the chromosomal heterozygotes [34],[37]. In that case there would be a narrow transition for the chromosomal fusion, but the distribution of the Y would be very similar to the neutral case (compare Figure 4, w_♀(FU)_<1 with the neutral case). However, if sexually antagonistic selection is operating, then these two clines will be displaced and the position on the Y cline relative to the fusion cline will indicate the relative strength of male beneficial and female deleterious effects on the Y. For example faster male evolution [44] would be supported if the Y cline were ahead of the fusion. This novel information on the forms of selection affecting young Y-chromosomes in natural populations has not previously been tapped.

A second indication of sexually antagonistic selection would be cline movement. In some cases it has proved possible to detect the actual movement of hybrid zones by repeated surveys or reviewing museum collections e.g. [45],[46]. In other cases the movement would be too slow, or held back by barriers to gene flow or gradients in population density [47]. It should still prove possible to identify slow or historical movement by surveys of other loci throughout the nuclear and cytoplasmic genome (for a review see [48]).

The realisation that surveys of sex chromosome hybrid zones can answer questions relating to the early evolution of sex chromosomes is exciting because such hybrid zones are already known and waiting to be analyzed. Examples include *Drosophila americana* [49], the morabine grasshopper *Vandiemenella (Warramaba) viatica* [25] and the grasshopper *Podisma pedestris* [34]. A great advantage of hybrid zone studies is that they involve wild populations [25] and some may represent snapshots of the actual establishment of a neo-XY system in nature, thus allowing the testing of theoretical predictions in biologically realistic conditions.

## Supporting Information

Dataset S1 Listing of the simulation which generated Figure 3. It is code which runs in R: the free software environment for statistical computing and graphics.
(0.04 MB RTF)
