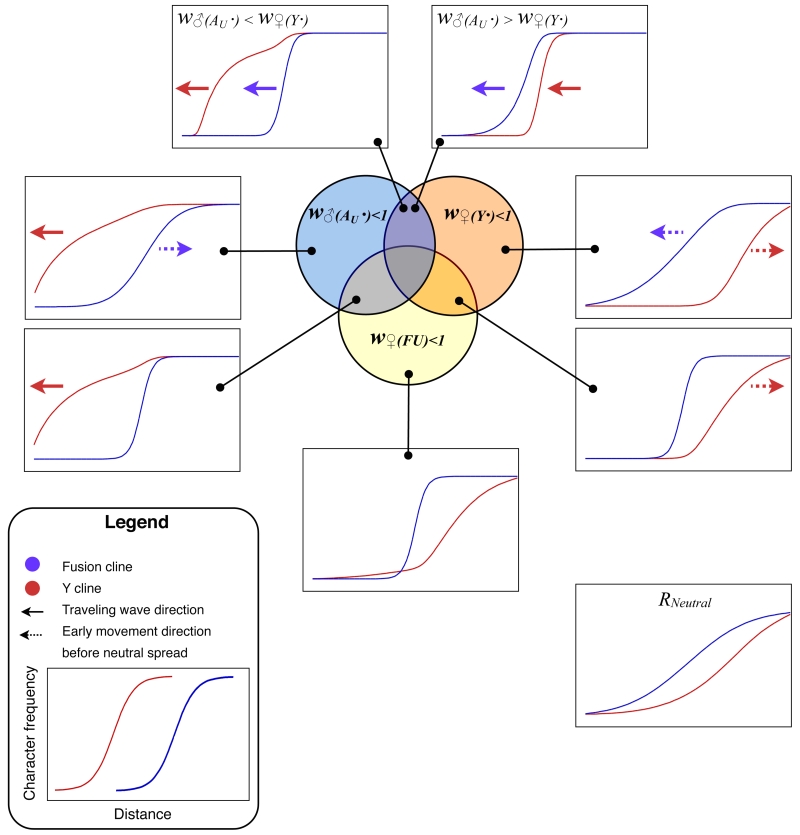# Correction: The Inexorable Spread of a Newly Arisen Neo-Y Chromosome

**DOI:** 10.1371/annotation/1b49686f-761d-4920-aba3-c8842d59b43a

**Published:** 2008-07-15

**Authors:** Paris Veltsos, Irene Keller, Richard A. Nichols

There was an error in Figure 4. In the top two panels, the inequality signs were reversed. The correct Figure 4 is available here:

**Figure:**