# Supplementary material for: The Inexorable Spread of a Newly Arisen Neo-Y Chromosome
Source: PLoS Genet. 2008 May 30;4(5):e1000082. doi: 10.1371/journal.pgen.1000082 (PMC2435400; doi:10.1371/journal.pgen.1000082)
Supplement: Dataset S1 — Listing of the simulation which generated Figure 3. It is code which runs in R: the free software environment for statistical computing and graphics. (0.04 MB RTF) [file pgen.1000082.s001.doc]

# evolve4 is the R function that carries out the simulation.
# Examples of its use are given below after the function definition.
# It should be possible to cut and paste the following code directly into R.


evolve4<-function(
	resolution=30,		# number of fitness values (0-1) to be evaluated
	m_dominance=1,	# dominance in males
	f_dominance=1,	# dominance in females
	Wfu=0.99,			# fitness of chromosomal heterozygotes
	Pa=0.001,			# initial frequency of the a allele
	Pf=0.004,			# initial frequency of the fusion
	numgens=1000)	# number of generations to simulate
{

# Utility functions for working with lists
Lsum<-function(a){
for (i in 2:length(a)) a[[1]]<-a[[1]]+a[[i]]
a[[1]]
}

LLprod <-function(L1,L2){
for (i in 1:length(L1)) L1[[i]]<-L1[[i]]*L2[[i]]
L1
}

Lmdiv <-function(L1,m){
for (i in 1:length(L1)) L1[[i]]<-L1[[i]]/m
L1
}

#Create a matrix of values for the fitness of the genotypes (aa,ab & bb) in males and females
mWbb<- matrix((0:resolution)/resolution,nrow=resolution+1,ncol=resolution+1, byrow=T)
mWab<- mWbb+(1-mWbb)*m_dominance
mWaa<- matrix(1,nrow=resolution+1,ncol=resolution+1)
fWaa<- matrix((0:resolution)/resolution,nrow=resolution+1,ncol=resolution+1)
fWab<- fWaa+(1-fWaa)*(1-f_dominance)
fWbb<- matrix(1,nrow=resolution+1,ncol=resolution+1)
# list the fitness in the same order as the genotypes
maleWs<-list(mWab,mWbb,mWaa,mWab,mWbb)
femWs<-list(fWbb,fWab*Wfu,fWbb*Wfu,fWaa,fWab,fWbb)

# create the lists to contain the gamete frequencies (initialized to HW proportions specified by initial frequencies)
Qa<-1-Pa
Qf<-1-Pf
z<- matrix(1,nrow=resolution+1,ncol=resolution+1)
eggs<-list(z*Pf,z*Qf*Pa,z*Qf*Qa)
names(eggs)<-c("F","Ua","Ub")

z<- matrix(0.5,nrow=resolution+1,ncol=resolution+1)
sperm<-list(z*Pf,z*Pa,z*Qa,z*Qf*Pa,z*Qf*Qa)
names(sperm)<-c("F","a","b","Ua","Ub")

# create the lists to contain the adult genotype frequencies
z<- matrix(0,nrow=resolution+1,ncol=resolution+1)
males<-list(z,z,z,z,z)
names(males)<-c("Fa","Fb","Uaa","Uab","Ubb")
females<-list(z,z,z,z,z,z)
names(females)<-c("FF","FUa","FUb","UUaa","UUab","UUbb")

for (gen in 1:numgens){
	# create male zygotes
	males$Fa<-	eggs$F*sperm$a
	males$Fb<-	eggs$F*sperm$b
	males$Uaa<-	eggs$Ua*sperm$a
	males$Uab<-	eggs$Ua*sperm$b	+eggs$Ub*sperm$a
	males$Ubb<-	eggs$Ub*sperm$b

	# create female zygotes
	females$FF<-	eggs$F*sperm$F
	females$FUa<-	eggs$F*sperm$Ua +eggs$Ua*sperm$F 
	females$FUb<-	eggs$F*sperm$Ub +eggs$Ub*sperm$F 
	females$UUaa<-	eggs$Ua*sperm$Ua
	females$UUab<-	eggs$Ua*sperm$Ub +eggs$Ub*sperm$Ua
	females$UUbb<-	eggs$Ub*sperm$Ub

	# do selection
	# multiply each zygote freq by fitness then normalize
	males<-LLprod(males,maleWs)
	males<-Lmdiv(males,Lsum(males))

	females<-LLprod(females,femWs)
	females<-Lmdiv(females,Lsum(females))

	# create the next generation of gametes
	sperm$F<- 	males$Fa *0.5		+males$Fb *0.5
	sperm$a<- 	males$Fa *0.5		+males$Uaa *0.5	+males$Uab *.25
	sperm$b<- 	males$Fb *0.5		+males$Ubb *0.5	+males$Uab *.25
	sperm$Ua<-	males$Uaa *0.5	+males$Uab *0.25
	sperm$Ub<-	males$Ubb *0.5	+males$Uab *0.25
	
	eggs$F<-		females$FF		+females$FUa * 0.5	+females$FUb *0.5
	eggs$Ua<-	females$FUa *0.5	+females$UUaa 	+females$UUab *0.5
	eggs$Ub<-	females$FUb *0.5	+females$UUbb 	+females$UUab *0.5
	}
list(sperm,eggs)
   
}

res<-30
#Run model example
results<-evolve4(resolution=30, m_dominance=.5, f_dominance=.5, Wfu=0.99, Pa=0.001, Pf=0, numgens=1000)

#2D plotting example
par(mfrow=c(2,2))
library(graphics)
contour(results[[1]]$a/(results[[1]]$a+results[[1]]$b), levels=c(0.001,.2,.4,.6,.8,.999))
contour(results[[2]]$F, levels=c(0.001,.2,.4,.6,.8,.999))
library(fields)

#3D plotting example
drape.plot(x=(0:res)/res,y=(0:res)/res,z=results[[1]]$a/(results[[1]]$a+results[[1]]$b), theta=-90,phi=45, zlab="Y frequency", add.legend=FALSE)
drape.plot(x=(0:res)/res,y=(0:res)/res,z=results[[2]]$F, theta=-90,phi=45, zlab="F frequency")


#2D graph of fusion for publication (note takes time to run due to high resolution)
results<-evolve4(resolution=500, m_dominance=.5, f_dominance=.5, Wfu=1, Pa=0.001, Pf=0.004, numgens=1000)
contour(results[[2]]$F, levels=c(0.001,0.999), lwd=1.5)


#2D graph of Y for publication (note takes time to run due to high resolution)
results<-evolve4(resolution=500, m_dominance=.5, f_dominance=.5, Wfu=0.99, Pa=0.001, Pf=0, numgens=1000)
contour(results[[1]]$a/(results[[1]]$a+results[[1]]$b), levels=c(0.001,0.999), lwd=1.5)
